# Comparative Genomic Analysis of Buffalo (*Bubalus bubalis*) NOD1 and NOD2 Receptors and Their Functional Role in *In-Vitro* Cellular Immune Response

**DOI:** 10.1371/journal.pone.0119178

**Published:** 2015-03-18

**Authors:** Biswajit Brahma, Sushil Kumar, Bidhan Chandra De, Purusottam Mishra, Mahesh Chandra Patra, Deepak Gaur, Meenu Chopra, Devika Gautam, Sourav Mahanty, Hrudananda Malik, Dhruba Malakar, Tirtha Kumar Datta, Sachinandan De

**Affiliations:** 1 Animal Genomics Lab, Animal Biotechnology Centre, National Dairy Research Institute, Karnal, 132001, Haryana, India; 2 Krishi Vigyan Kendra, Bhaderwah, SKUAST-Jammu, Jammu, India; 3 Department of Pharmaceutical Research and Education Center, Kazan (Volga Region) Federal University, Kazan, Russia; University of São Paulo, BRAZIL

## Abstract

Nucleotide binding and oligomerization domain (NOD)-like receptors (NLRs) are innate immune receptors that recognize bacterial cell wall components and initiate host immune response. Structure and function of NLRs have been well studied in human and mice, but little information exists on genetic composition and role of these receptors in innate immune system of water buffalo—a species known for its exceptional disease resistance. Here, a comparative study on the functional domains of NOD1 and NOD2 was performed across different species. The NOD mediated *in-vitro* cellular responses were studied in buffalo peripheral blood mononuclear cells, resident macrophages, mammary epithelial, and fibroblast cells. Buffalo NOD1 (buNOD1) and buNOD2 showed conserved domain architectures as found in other mammals. The domains of buNOD1 and buNOD2 showed analogy in secondary and tertiary conformations. Constitutive expressions of NODs were ubiquitous in different tissues. Following treatment with NOD agonists, peripheral lymphocytes showed an IFN-γ response along-with production of pro-inflammatory cytokines. Alveolar macrophages and mammary epithelial cells showed NOD mediated *in-vitro* immune response through NF-κB dependent pathway. Fibroblasts showed pro-inflammatory cytokine response following agonist treatment. Our study demonstrates that both immune and non-immune cells could generate NOD-mediated responses to pathogens though the type and magnitude of response depend on the cell types. The structural basis of ligand recognition by buffalo NODs and knowledge of immune response by different cell types could be useful for development of non-infective innate immune modulators and next generation anti-inflammatory compounds.

## Introduction

The germ line encoded pattern recognition receptors (PRRs) are innate immune sentinels that recognize conserved microbe-associated molecular patterns (MAMPs) and initiate host response against microbial invasion. Nucleotide-binding and oligomerization domain (NOD)-like receptors (NLRs) were identified as intracellular PRRs in human about 15 years ago [1–3]. These receptors were earlier thought to be of vertebrate specific, but recent identification of NLR genes in the genomes of the sea urchins [4] and cnidarians suggests that they have an ancient origin. NLR proteins have tripartite domain architecture consisting of a C-terminal leucine-rich repeat (LRR) domain that senses microbial signature, a central NOD or NACHT domain, and a variable N terminal effector domain containing a DEATH fold (PYD, CARD, BIR, and unclassified) [5,6]. Based on the type of N-terminal effector domain, a recent nomenclature system subdivides the NLR family into four subfamilies (NLRA, NLRB, NLRC and NLRP) [7]. Besides sensing MAMPs, NLRs are also involved in detecting endogenous non-microbial “danger” or ‘stress’ signals. Mutations in the NLR genes are associated with chronic inflammatory disorders such as Crohn’s disease (NOD2), Muckle—Wells syndrome (NLRP3), atopic disorders (NOD1) and vitiligo (NLRP1) [8]. NOD1 and NOD2 are the prototypical NLRs belonging to the NLRC subgroup with a single and tandem N-terminal CARD domain(s), respectively [9]. Both NOD1 and NOD2 sense bacterial peptidoglycans, but specific ligand moieties are different for these receptors. NOD1 recognizes γ-D-glutamyl-meso-diaminopimelic acid (iE-DAP), a dipeptide produced by most gram-negative bacteria and a few gram-positive bacteria. NOD2 senses the muramyl dipeptide (MDP) peptidoglycan motif present in nearly all gram-negative and gram-positive bacteria [10–14]. After sensing the ligand, the NATCH domain of the receptors oligomerizes and downstream signaling cascade is initiated through a homotypic CARD—CARD interaction between NOD1/NOD2 and the kinase RICK (also called RIPK2/RIP2) [15–17]. Ubiquitination plays an important role for the activation of RICK, successive signaling, and activation of the TAK1 complex. RICK promotes Lys63-linked ubiquitination of NEMO, allowing the recruitment of TAK1 that phosphorylates IKK-β, leading to degradation of the nuclear factor kappa-B (NF-κB) repressor IkB. This allows NF-κB translocation to the nucleus and initiation of transcription of pro-inflammatory genes. Recently, NOD1 and NOD2 are also recognized as the drivers of autophagosome formation during infection through a pathway that is independent of the adaptor RICK and NF-κB [18]. The NOD proteins recruit autophagy protein ATG16L1 that interacts with a conjugate of ATG5 and ATG12. The complex promotes conversion of LC3 to LC3-II, leading to the autophagosome formation [18].

Buffaloes (*Bubalus bubalis*) are well-known for their exceptional disease resistance [19] and can thrive in wet grasslands, swamps and harsh tropical and sub-tropical climates of Indian sub-continent, Mediterranean regions, Caribbean, Africa and South America. Buffaloes are less susceptible to tick-borne diseases [20] and severities of diseases such as trypanosomiasis, tuberculosis, brucellosis, rinderpest and piroplasmosis are less deleterious in buffalo compared to cattle [21]. While considerable research on the precise mode of NLR activation and signaling cascades has been done on human and mice, little is known about the contribution of NLRs in the innate resistance of other species, *e*.*g*. bovine, ovine, and porcine. In this study, we have attempted to address this gap by analyzing the sequence and modeled structures of buffalo NOD receptors; their expression patterns in different tissues; and the *in-vitro* response of different cell types upon activation of these receptors.

## Materials and Methods

### Ethics Statement

Buffalo tissues were collected from the Municipal Slaughter House, New Delhi, India with permission for research use. The slaughter house follows all the ethical and humane standards for animal slaughter and is regulated by norms of Government of India. National Dairy Research Institute (NDRI), as another government organization, is permitted to collect animal tissues for research use. Blood samples were collected from healthy female Murrah buffalo calves less than one year of age maintained under standard management at the experimental animal herd of NDRI. Permission was taken from Institutional Animal Ethics Committee (IAEC) of NDRI. The samples were collected by skilled technicians after proper restraining of animals under the supervision of a veterinary officer present at the cattle yard, NDRI. For RNA isolation from blood, five samples were collected. For PBMC isolation, three blood samples were collected. No animal was specifically slaughtered for this research.

### Materials

Unlabelled goat anti-bovine polyclonal antibodies against NOD1, NOD2, NF-κB, and FITC-labeled rabbit anti-goat IgG were obtained from Santa Cruz Biotechnology Inc (CA, USA). Unlabeled mouse anti-bovine monoclonal antibody against β-actin, horseradish peroxidase-labeled rabbit anti-goat IgG and FITC-labeled rabbit anti-mouse IgG were purchased from Sigma-Aldrich (St. Louis, MO, USA). Taq DNA polymerase, 10X buffer, dNTP were purchased from New England Biolab (MA, USA). Fermentas Maxima SYBR Green qPCR Master Mix (2) was obtained from Thermo Fisher Scientific Inc. (PA, USA). γ-D-Glu-mDAP (iE-DAP), MDP, γ-D-Glu-Lys (iE-Lys) and MDP control (D-alanine instead of L-alanine) were obtained from Invivogen (San Diego, CA). RPMI-1640, Dulbecco Modified Eagle’s medium (DMEM), Trypsin-EDTA, foetal bovine serum (FBS), ITS Liquid Media Supplement (100×), hydrocortisone and Epidermal Growth Factor (EGF) were obtained from Sigma-Aldrich (St. Louis, MO, USA). L-Glutamine (Glutamax 100×) was purchased from Invitrogen corp., (Carlsbad, CA, USA). bGM-CSF and bIL-4 were purchased from Abd Serotec (Oxford, UK). Penicillin-G and streptomycin were obtained from Amresco (Solon, OH, USA). Plasticwares used for cell cultures were from Nunc (Roskilde, Denmark). Filters (Millex GV. 0.22 μm) were purchased from Millipore Pvt. Ltd., (Billerica, MA, USA). All other reagents were of analytical grade.

### Isolation and maintenance of cells

Blood samples from healthy young calves (under one year age) maintained at experimental herd of National Dairy Research Institute, were collected in heparin coated vacutainers. The buffy coat was isolated by centrifugation (300 g for 8 min.) and diluted with Dulbecco phosphate buffer saline (DPBS). Lymphocyte-enriched peripheral blood mononuclear cells (PBMCs) from buffy coats were isolated by density gradient centrifugation using Histopaque-1077 (Sigma-Aldrich, MO, USA). The interphase fraction was collected and washed twice with DPBS. The pellet was resuspended in serum free RPMI-1640 medium supplemented with L-Glutamine (2 mM), Penicillin-G (10000 U/ml) and streptomycin (100 μg/ml). Viable cell count was determined by the trypan blue exclusion method. Cells with viability >98% were plated in non-treated polypropylene microtitre plate (Greiner Bio-one, NC, USA) at a concentration of 1 10^6^ cells/ml for *in-vitro* experiments.

For isolation of alveolar macrophages, lung tissues were obtained from New Delhi Municipal slaughter house. Bronchoalveolar lavage was obtained by washing the bronchus and alveoli with Hanks’ balanced salt solution (HBSS) supplemented with Penicillin-G (10000 U/ml) and streptomycin (100 μg/ml). The lavage was passed through a sterile muslin cloth and centrifuged at 300g for 5 min. The pellets were washed twice with HBSS before suspending in complete RPMI-1640 medium supplemented with 10% FBS, L-Glutamine (2 mM), Penicillin-G (10000 U/ml) and streptomycin (100 μg/ml). The cell suspensions were then placed onto 100 mm Nunclon surface tissue culture disks to facilitate adherence of macrophages to the surface. After 2 h the adhered cells were washed twice with DPBS. The cells were harvested and viability was determined by trypan blue staining. The macrophages were plated onto four well plates at a concentration of 10^6^ cells/ml and cultured overnight in serum free RPMI-1640 medium. For maintenance of cells, macrophages were cultured in complete RPMI-1640 medium supplemented with 10% FBS, recombinant bGM-CSF (10 U/ml) and bIL-4(10 U/ml).

Buffalo Mammary Epithelial Cell (buMEC) line, previously established by our colleagues [22] was grown in Dulbecco Modified Eagle’s medium (DMEM) supplemented with 10% FBS, 10 ng/ml EGF, 10 μg/ml ITS liquid media supplement (containing 1.0 mg/ml insulin, 0.55 mg/ml transferrin and 0.5 μg/ml sodium selenite), 1 μg/ml hydrocortisone, 100 U/ml penicillin, 5 μg/ml streptomycin and 50 ng/ml amphotericin. The cells were seeded at a density of 10^5^ cells/well and cultured in four well plates for three days to attain 70–80% confluence.

Primary culture of foetal skin derived fibroblast cells were prepared by a previously described method [23]. The cells were maintained in complete Dulbecco Modified Eagle’s medium (DMEM) supplemented with 10% FBS, 25mM HEPES, 10 ng/ml EGF, L-Glutamine (2 mM), Penicillin-G (10000 U/ml) and streptomycin (100 μg/ml).

### Inductive expressions of NOD1 and NOD2 in different cells types

The inductive expression of NOD1, NOD2 and associated genes were studied in four types of cell population *viz*. PBMCs, macrophages, epithelial and fibroblast cells. Lymphocyte rich PBMCs over purified B or T lymphocytes was preferred as pure lymphocyte population requires assistance of antigen presenting cells (APCs) or soluble mediators released from APCs for induction of a TLR or NLR response [24–25]. Epithelial and fibroblast cells are frequently exposed to bacteria (*e*.*g*. intestinal, bronchial epithelia and skin), and therefore, we considered it pertinent to study the NOD mediated response of these cells to bacterial MAMPs. For treatment with agonists, cells were maintained in serum free medium at 37°C at least for 6 h. Two known agonists iE-DAP (10 μg/ml) and MurNAc-L-Ala-D-isoGln, also known as MDP (10 μg/ml) were used to stimulate NOD1 and NOD2, respectively. Also, iE-Lys and MDP-control (D-alanine instead of L-alanine) were used as sham controls (shCONTROL) for iE-DAP and MDP, respectively. All experiments were performed with four replicates for each treatment or control group.

### Determination of NF-κB translocation to nucleus

The translocation of NF-κB from cytoplasm to nucleus was observed by immunocytochemistry. Cells were grown on sterilized glass coverslips placed in 12-well tissue culture plates. The coverslips following agonist treatments were taken out, washed with DPBS, and placed on a 35 mm tissue culture disks. Cells were fixed by incubating with freshly prepared 3.7% (v/v) formaldehyde in PBS for 20 min. Cells were treated with permeabilization buffer (0.5% Triton X-100, 0.2 μg/ml EDTA in 1 PBS) for 10 min. followed by three rinses with PBS. Non-specific sites were blocked by with blocking reagent (1% BSA, 10% FBS-PBS) at 4°C. Cells were then incubated with primary antibodies of NF-κB for 2 h at RT, washed three times with PBS, and incubated with FITC conjugated rabbit anti-goat IgG antibody. Counterstaining was done by DAPI. The cover slips were washed, mounted on slides with anti-fade DPX. Fluorescent micrographs of cells were taken at 200 magnification in a microscope (Olympus BX51 fitted with DP71 camera) with a fluorescence illuminator system. Distinctive staining of nucleus was considered as positive for NF-κB translocation to nucleus, whereas, staining of cytoplasm with a clear zone at the position of nucleus were regarded as negative. Cells from at least five microscopic fields were counted and assigned a score as follows: 0–10% as negative (-); 11–20% as “+”, 21–40% as “++”, 41–60% as “+++”, and >60% as “++++”.

### PCR amplification

Primers for full length gene and real time PCR were designed by aligning gene sequences of several mammals including cow, pig, mouse, buffalo, and human (S1–S2 Tables). Total RNA from tissues/ cells were isolated by TRIzol method by following manufacturer’s instructions. About 1μg of RNA was used for cDNA preparation (Superscript III cDNA synthesis kit; Invitrogen, USA). All PCR amplifications were performed in 25 μl reaction volume. Each reaction contained 2.5 μl 10× buffer, 200 μM of dNTPs, 0.5 μl of each primers (10 pmol), 0.5 units of Taq DNA polymerase and nuclease free water to bring the total volume to 25 μl. Around 100 ng of cDNA was used as template. Thermal cycling parameters were optimized for different fragments/gene with a touchdown protocol (S3 Table). The PCR products were resolved on a 1.5% agarose gel. The PCR products were cloned on to pTZ57R/T vector, plasmids were screened using PCR, and plasmids containing desired gene fragments were custom sequenced.

### Real time PCR (qRT-PCR)

Wherever applicable, equal amount of RNA (quantified using Qubit High Sensitivity RNA assay kit and Qubit fluorometer, Invitrogen) were used for cDNA preparation (Superscript III cDNA synthesis kit; Invitrogen). All qRT-PCR reactions were conducted on a Light Cycler 480 II Real-Time PCR machine (Roche Diagnostics, USA). Each reaction consisted of 2 μl cDNA template, 5 μl of 2X SYBR Green PCR Master Mix 0.25 μl each of forward and reverse primers (10 pmol/μl) and nuclease free water for a final volume of 10 μl. Each sample was run in duplicate. Analysis of real-time PCR (qRT-PCR) was performed by delta-delta-Ct (ΔΔCt) method [26].

### Western Blotting

About 100μg of total proteins, extracted from cell lysate of representative experimental groups, were resolved by 10% SDS-PAGE gel at constant voltage of 40 V. Proteins on the gel were transferred to Immobilon-P polyvinylidenedifluoride (PVDF) membranes (Millipore, Bedford, MA). Blocking was done with 5% (w/v) non-fat dried milk (prepared in 20 mM Tris, 150 mM NaCl, pH 7.6; 0.1% (v/v) Tween-20) for overnight at 4°C. Primary antibodies were used at the following dilution: 1:250 anti-NOD1, 1:250 anti-NOD2 and 1:5000 anti-β-Actin. Membranes were washed three times and incubated with secondary antibody (dilution 1:50000 for anti-goat IgG and 1:70000 for anti-mouse IgG). The target protein was detected by chemiluminescence (Immobilon western chemiluminescent HRP substrate; (Millipore, Bedford, MA) captured on X-ray films. The specificity of NOD1 and NOD2 antibodies was confirmed previously with western blots (S1 Fig.).

### Sequence analysis and homology modeling

The amino acid sequences of buNOD1 and buNOD2 were translated from respective nucleotide sequences identified in this study (GenBank ID: KJ767654-KJ767655). Ortholog sequences of fish, rodent, human, and other ruminants were retrieved from NCBI (S4 Table) and putative conserved domains and critical binding site residues within buNOD1 and buNOD2 were identified using Domain Enhanced Lookup Time Accelerated (DELTA) BLAST, Conserved Domain Database (CDD) of NCBI and UniProtKB. Multiple sequence alignments were performed in CLUSTALW2 web server. Phylogenetic analysis was carried out in MEGA 5.2 software [27]. Estimates of evolutionary divergence among sequences were conducted by calculating pair-wise distances using the JTT matrix-based model [28]. The results were exported to R program [29] and the values were plotted as heat-map. Homology modeling was done by searching homologous templates for buffalo sequences using DELTA BLAST tool against the known protein structures available in PDB. Based on maximum identity, the tertiary structures were predicted using advance modeling protocol of MODELLER 9.11 [30]. The following templates were used for modeling different domains of buNODs: buNOD1-CARD (PDB ID: 2NZ7), buNOD2-CARD-I and CARD-II (PDB ID: 1DGN), NACHT domains of buNOD1 and buNOD2 (PDB ID: 4KXF), buNOD1-LRR (PDB ID: 2BNH) and buNOD2-LRR (PDB ID: 3TSR). The resultant models were ranked based on Discrete Optimized Potential Energy (DOPE) score and the models with lowest DOPE scores were selected for further study. Stereochemical quality assessment and model validation were performed by VERIFY3D [31], ERRAT [32], and ProQ[33] programs (S5 Table).

### Statistical analysis

Statistical analyses were carried out in SYSTAT v12.02 software (SYSTAT Software Inc.) Analysis of variance (ANOVA) was used to test between groups and hour intervals. Fischer’s restricted least significant differences criterion was used to maintain the a priori type I error rate of 0.05.

## Results

### Comparative genomic analysis and evolutionary perspective of buNOD1 and buNOD2

**Fig 1:**
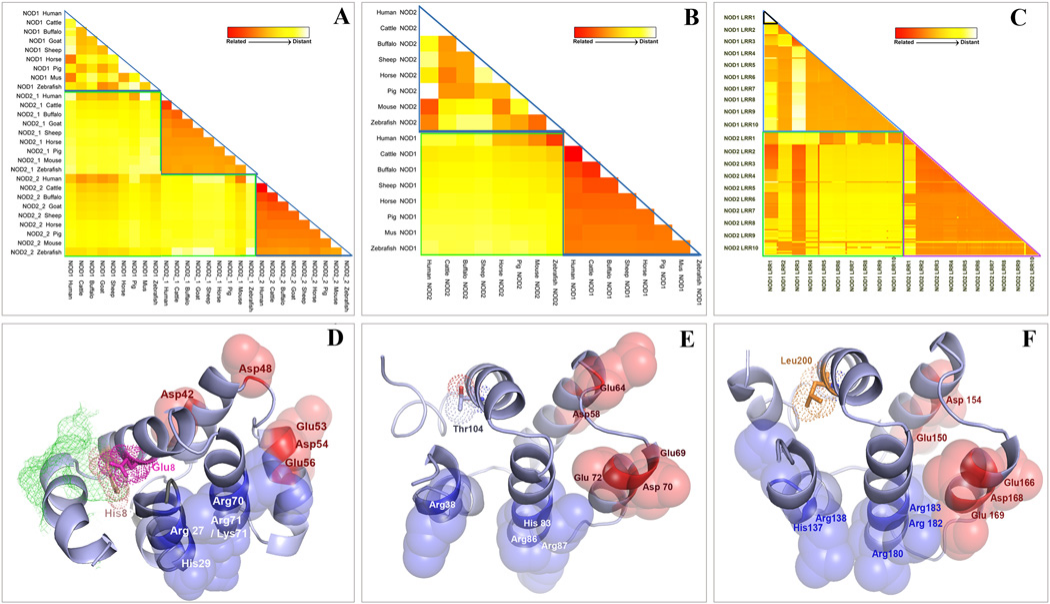
Amino acid conservation of different domains of NOD1 and NOD2 among different species. Estimates of evolutionary divergence among sequences were conducted by calculating pair-wise distances using the JTT matrix-based model. The zone marked with green lines spans comparison of respective region of NOD1 and NOD2 in different species. The blue or violet lines span the zones forinter-species comparison of a region specific to either NOD1 or NOD2. (A) The map indicated NOD2 CARDs are well conserved among different species, NOD1 CARD is less conserved and there were little similarities amongst the different CARDs in the species under consideration. (B) NOD1 NACHT is well conserved among different species compared to NOD2 NACHT. There was distant correlation amongst the NACHT domains of NOD1 and NOD2 in species under consideration. (C) Distances among LRRs of NOD1 and NOD2 of different species. Each small triangle (example marked with black lines) or square represent nine species under study (for clarity of the figure, species names have not shown). (D-F) Cartoon representations showing conserved amino acids responsible for forming basic (blue) and acidic (red) patches on three cards. Residues D42, D48, E53, D54, and E56 have been implicated for CARD-CARD interaction of NOD1 and RICK. Ubiquitinylation sites E85 (pink) was conserved Y89 was substituted with histidine that was found to be was buried by side chains of L93, Y96 and L100. Residues R38 and R86 required for NOD2 CARD-I and RICK CARD interaction were also conserved in buffalo. Residues implicated for ubiquitinylation in NOD2 CARDs have been also shown with their electron density spheres.

Core building blocks of NLRs, such as, NB-ARC, NACHT, DD, and LRR, exist in eubacteria, archaebacteria and fungi, but formation of NLRs by the fusion of domains had first taken place in metazoans (S2 Fig.). Among metazoans, mammals harbor less number of NLR genes compared to lower vertebrates. Orthologs of mammalian NOD1 and NOD2 can be traced in zebrafish, though NOD2 gene appears to be lost in the amphibian and avian lineages [34]. Analysis of amino acid sequence showed characteristic domain organization of buNOD1 (CARD-NACHT-LRRs) and buNOD2 (CARD-I-CARD-II-NACHT-LRRs). Buffalo NODs showed fair (56.02%) and high (86.26%) levels of amino acid conservation with human NOD1 and NOD2, respectively. However, little homology was observed between NOD1 and NOD2 for the species considered in the study including buffalo (9.38%) and human (14.83%). Analysis of respective domains (CARDs, NACHTs and LRRs) of NOD1 and NOD2 across different species supported a distant relationship of these genes (Fig. 1a-c). Comparison across different species indicated a heterogeneous relationship among CARDs of NOD1 and NOD2. This is explicable since primary mode and interfaces of CARD-CARD interactions with RICK are different for NOD1 and NOD2. It has been shown that residues on acidic patch formed by helices 2 and 3 of NOD1-CARD interact with basic residues of RICK CARD [35–36]. A different mode involving basic residues of NOD2 and acidic residues of RICK has been shown responsible for CARD-CARD interaction of NOD2 and RICK. [37]. Also, NOD1-CARD favors a transition from monomeric to homodimeric form in basic environment by helix swapping and interchain disulfide bond formation through Cys39 residue [38–39]. Corresponding with previous studies [36], this residue (Cys39) was found conserved in buffalo as well. Residues Arg35, Asn36, Ala94, Tyr97, Leu100, Arg101, Trp103 and Leu104 that primarily contributes to dimeric interface of NOD1-CARD [36], were also conserved in buffalo. Residues of NOD1-CARD (Leu40, Val41, Asp42, Leu44, Asp48, Glu53, Asp54, Glu56 and Arg69), implicated in RICK binding and signal transduction [34, 36], were conserved across the species with exceptions of ruminant specific (cattle, buffalo and sheep) V41L and mouse specific D48G replacements. Mutation studies have shown reduced signaling ability of NOD1-CARD with V41A and V41Q replacements, but no apparent change in signaling and RICK binding activities was observed with D48K replacement [36]. Among the residues responsible for ubiquitin binding during NOD1-RICK interaction [40], Glu84 was conserved in mammals, but Tyr88 was substituted with other bulky residues, such as histidine in buffalo and other ruminants and phenylalanine in porcine, reported in earlier study as well [36]. Homology modeling of buNOD1 CARD showed that Glu84 was partially solvent accessible on the surface, but His88 was buried by the side chains of Leu93, Tyr96, and Leu100 (Fig. 1c). The two CARDs of NOD2 shared little homology between them (14.7% in human and buffalo), but across the species, conservation of residues was found for both the CARDs (Fig. 1a). The tandem CARDs of NOD2 are engaged in an intramolecular interaction, although residues participating in such interaction have not been identified yet [37]. Residues (Arg38 and Arg86 of NOD2 CARD-I) implicated for interaction with RICK were conserved across the species agreeing with a previous observation [34]. Among designated residues of ubiquitin binding [40], Leu200 of NOD2 CARD-II were conserved in mammals, but Ile104 of NOD2 CARD-I was replaced by valine in mouse, zebrafish, or threonine in cattle, buffalo, and sheep. The overall analysis suggests conservation of critical residue within an individual CARD across species, but little homology among the three CARDs (NOD1CARD-I, NOD2CARD-I and NOD2 CARD-II) indicated that they are unrelated.

Like CARDs, NACHTs domains of NODs revealed little homology, but patches of conservation were found in important regions. Inter-species comparison showed a conserved nature of NOD1-NACHT in different species but NOD2-NACHT was much diverse among species under study (Fig. 1b). The motifs associated with ATP-binding, viz. Walker A/P-loop, Walker B, and Sensor [5], were identified in both NACHTs (S3 Fig.). The Walker A motif showed a consensus of G-E/D-A-G-x-G-K-S-T, where the central lysine residue is important for interaction with phosphate moiety of ATP [41]. Like other NTPases, the P-loop was preceded by a β-strand and is followed by an α-helix. The Walker B-motif (consensus LxhhDGxDEx; h = hydrophobic residue, x = any amino acid) consisted of a single conserved aspartate at the C-terminus of a β-strand and two conserved aspartate/glutamate at close proximity. The first aspartate is commonly involved in anchoring an Mg^2+^ion, whereas, the adjacent aspartate/glutamate usually provides the catalytic carboxylate for NTP-hydrolysis [42]. Sensor-1 region was located immediately after fourth β-strand (fifth for NOD2) and showed a conserved arginine residue that contacts the γ-phosphate of the bound NTP and is thought to discriminate between ATP and ADP [42]. The sensor 2 motif seems to be absent in NACHT domains of NODs. The GxP motif showed a conserved proline that is required for interaction with the adenine group of ATP, but the glycine residue was different in both the NACHT domains. It was found that despite low sequence homology between NACHT domains of NOD1 and NOD2, there was little difference in the secondary structures (S3 Fig.), indicating the overall tertiary conformation of NACHTs could be similar.

**Fig 2:**
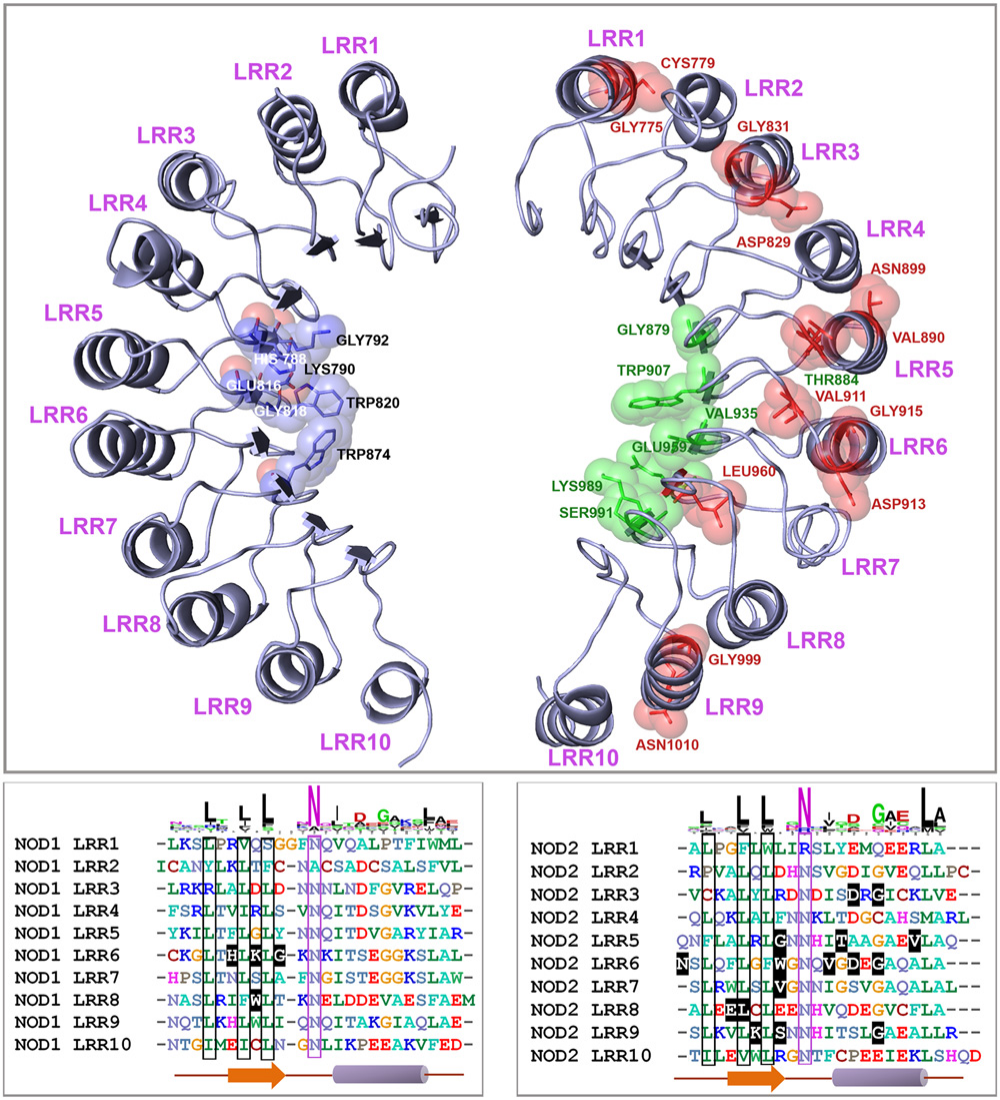
Comparison of LRR domains of buNOD1 (left panel) and buNOD2 (right panel). Ten putative LRRs were identified in both NOD1 and NOD2 sequences of buffalo. Conserved amino acids responsible for ligand binding and interaction were mapped to models and highlighted in the alignment. Residues indicated in models but not shown in alignment were located in the intermediate loop region of turn and helix. Residues H788, K790, G792, E816, G818, W820, W874 of NOD1 LRRs, responsible for iE-DAP binding, formed a cluster at central concave face. Residues important for NOD2’s response to MDP (highlighted green and red spheres) were located in β-sheet/ β-sheet turns, loop regions as well as in the α-helices, while residues involved in MDP binding (red) were confined to the β-strand/ β-turn motif at the central concave face of LRR.

Using UniprotKB database and earlier studies [43–44], we identified 10 LRRs each in buNOD1 and buNOD2. The LRRs of buNODs showed a consensus LxLxxNxL motif, where L = Leu, Val, Ile, Phe; N = Asn, Ala, Arg; x = any amino acid (Fig. 2). Corresponding LRRs of NOD1 and NOD2 showed heterogeneity except NOD2 LRR2 that shared a little homology with LRR1 and LRR3 (Fig. 1c). Homology models of LRRs of buNOD1 and buNOD2, based on the X-ray structure of the ribonuclease inhibitor exhibited a characteristic horseshoe-shaped structure with parallel beta sheets forming the concave groove for ligand binding (Fig. 2). Despite several studies have indicated that NOD1 and NOD2 may directly interact with their respective agonists [45–47], this is yet to be established conclusively [48]. Studies have indicated that mutation in NOD1 LRRs residues His788, Lys790, Gly792, Glu816, Gly818, Trp820, and Trp874 are associated with impaired receptor functions [49]. These residues were conserved in buffalo, but a few were variable in other species. His788 was replaced by a tyrosine in horse, cysteine in pig, and threonine in zebrafish. An isotypic replacement of Glu816 (E816D) was also observed in mouse and zebrafish. The mapping of the residues onto modeled NOD1 LRR domain showed their position on the β-sheet/turns, forming a cluster at central concave face (Fig. 2, left panel). Residues of NOD2 LRR, important for NOD2 responsiveness to MDP [44], were mapped on to β-sheets, loop regions, and α-helices of the LRRs (Fig. 2, right panel). However, residues implicatedin MDP binding (Gly879, Trp907, Val935, Glu959, Lys989 and Ser991) [44] were confined to the β-strand/ turn motifs at the central concave face of LRR domain. All these critical residues were conserved in buffalo, except Thr899 that was replaced by asparagine in ruminants (cattle, sheep, and goat), and by glutamine in zebrafish. Also a Val890Gln replacement was observed in zebrafish. It is intriguing that despite little homology amongst LRRs of NOD1 and NOD2, the residues predicted for ligand binding were of similar type and were mapped on identical positions of the models (*e*.*g*. Gly792–Gly879, Trp820–Trp907, Gln816–Gln959, and Lys790–Lys989 of LRRs of NOD1 and NOD2, respectively). This similarity at ligand binding site is indicative of a conserved mechanism of ligand recognition by NOD1 and NOD2 LRRs, though precise significance remains a subject of further research considering that the receptors have different ligand specificity. Taken together, our analyses suggest that despite low sequence identity, the respective domains of NOD1 and NOD2 share similarities at tertiary conformation, indicating basic principles of MAMP recognition and signaling could be similar for the receptors.

### Constitutive expression of NLRs

**Fig 3:**
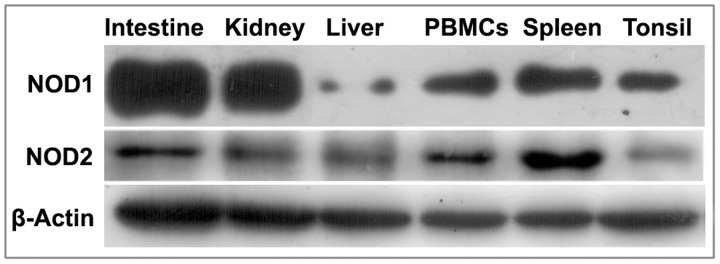
Constitutive expression and localization pattern of buffalo NOD proteins. Constitutive protein expression of buffalo NOD1 and NOD2 in different tissues detected by Western blot using equal amount of proteins from the tissues.

The constitutive expression pattern of NODs was investigated in freshly isolated peripheral blood mononuclear cells (PBMCs), spleen, and tonsils, intestine, kidney and liver. Intestine and kidney showed abundant expression of NOD1 (Fig. 3). Spleen, tonsil and PBMCs also showed fair expression of NOD1. Unlike NOD1 that was ubiquitously expressed in almost all the tissues studied, the expression of NOD2 was mainly observed in spleen and to a lesser extent in intestine and PBMCs. Kidney and tonsil showed very low amount of NOD2 expression. Liver showed negligible expression of both NOD1 and NOD2 proteins. It is generally considered that the expression of NOD2 is restricted to haematopoietic compartment, while NOD1 expression could be found in cells of both haematopoietic and non-haematopoietic origin [49]. Among haematopoietic cells, macrophage, monocytes, granulocytes, and dendritic cells express high level of NOD2 [50], and lower level of NOD2 expression has been found in T lymphocytes [13] and B lymphocytes [51]. However, expression of NOD2 has also been confirmed in intestinal epithelial cells [52–53], hepatocytes [54], paneth cells [55], lung kidney and oral epithelial cells [56–58]. While our study concurs to the many earlier findings reporting constitutive expression patterns of NODs in human and mice tissues, it was perceived that NOD2 expression is more restrictive in liver and kidney. In brief, constitutive expression of NODs was not restricted to primary immune organs and was ubiquitous to different types of tissues.

### NOD mediated cellular response following iE-DAP and MDP treatment

The cellular events following stimulation of NOD1 and NOD2 receptors by iE-DAP and MDP ligands were studied in different types of cells *viz*. PBMC, macrophages, mammary epithelial cells and fibroblasts. The type and duration of responses following agonist induced expression of NOD1 and NOD2 were varied and dependent upon cell types.

### Agonist induced cellular response of buffalo PBMCs

**Fig 4:**
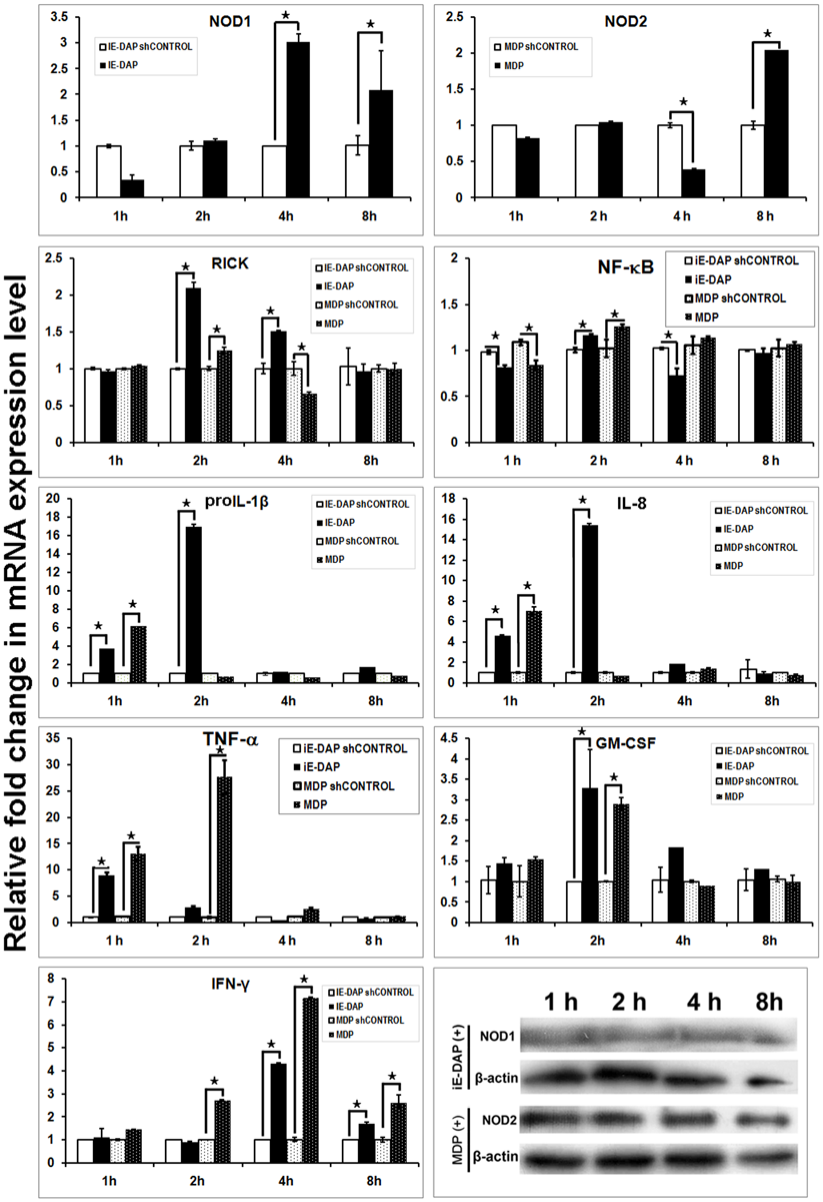
Inductive mRNA expression of NOD1, NOD2, downstream adapter (RICK), and effector (interleukins and interferons) in iE-DAP and MDP treated PBMCs. X-axis represents the time intervals following agonists addition, Y axis shows relative fold change in mRNA expression of genes over respective controls (sh-controls). Columns indicated with asterisks (*) differ significantly (p<0.05) from their respective controls. Protein expression of NOD1, NOD2 and β-actin in treated cells over different time intervals has been shown also.

Buffalo PBMCs (buPBMCs) showed a significant increase in mRNA expression of NOD1 (4–8 h) and NOD2 (8 h) following exposure to iE-DAP and MDP, respectively (Fig. 4). A coherent protein expression of NOD1 and NOD2 was observed, though protein expression of NOD1 was less apparent in PBMCs. Increased mRNA expression of RICK, was observed following iE-DAP (2–4 h) and MDP (2 h) treatments. Agonist treated PBMCs showed elevated mRNA levels of pro-inflammatory cytokines proIL-1β, IL-8 and TNF-α, but the response ceased after 1 h in MDP treated cells. We found no significant alteration in the mRNA expression of ATG-16L or autophagy associated genes (S4 Fig.), which suggested that pro-inflammatory cytokine production in MDP treated PBMCs was not likely inhibited by ATG-16L [59]. A significant observation in both MDP and iE-DAP treated buPBMC was the induction of an IFN-γ response, the amplitude and duration of which were more evident in MDP treated cells. This response was specific to PBMCs from young calves but was not apparent in PBMCs of mature animals (data not shown). It is noteworthy that like other ruminants, young buffalo calves have a high percentage (~75%) of circulating γδ T cells [60]. Elevated IFN-γ level following MDP treatment has been observed in human γδ T cells [61]. Associated with IFN-γ secretion, a significant increase in the GM-CSF transcripts was also observed in both MDP and iE-DAP treated cells. The increased GM-CSF level following MDP treatment was consistent with the earlier findings [62], but the study showed increased IFN-γ and GM-CSF levels could be associated with iE-DAP stimulation as well. Together, NOD mediated immune response in buPBMCs had two basic differences than that had been observed earlier for human B or T lymphocytes, tonsilar mononuclear cells, neutrohils and eosinophils [24, 63–65]. First, the duration of pro-inflammatory cytokine response was brief, especially in MDP treated cells and second, the cells showed an IFN-γ response following exposure to NOD agonists.

### Agonist induced cellular response of buffalo alveolar macrophages

**Fig 5:**
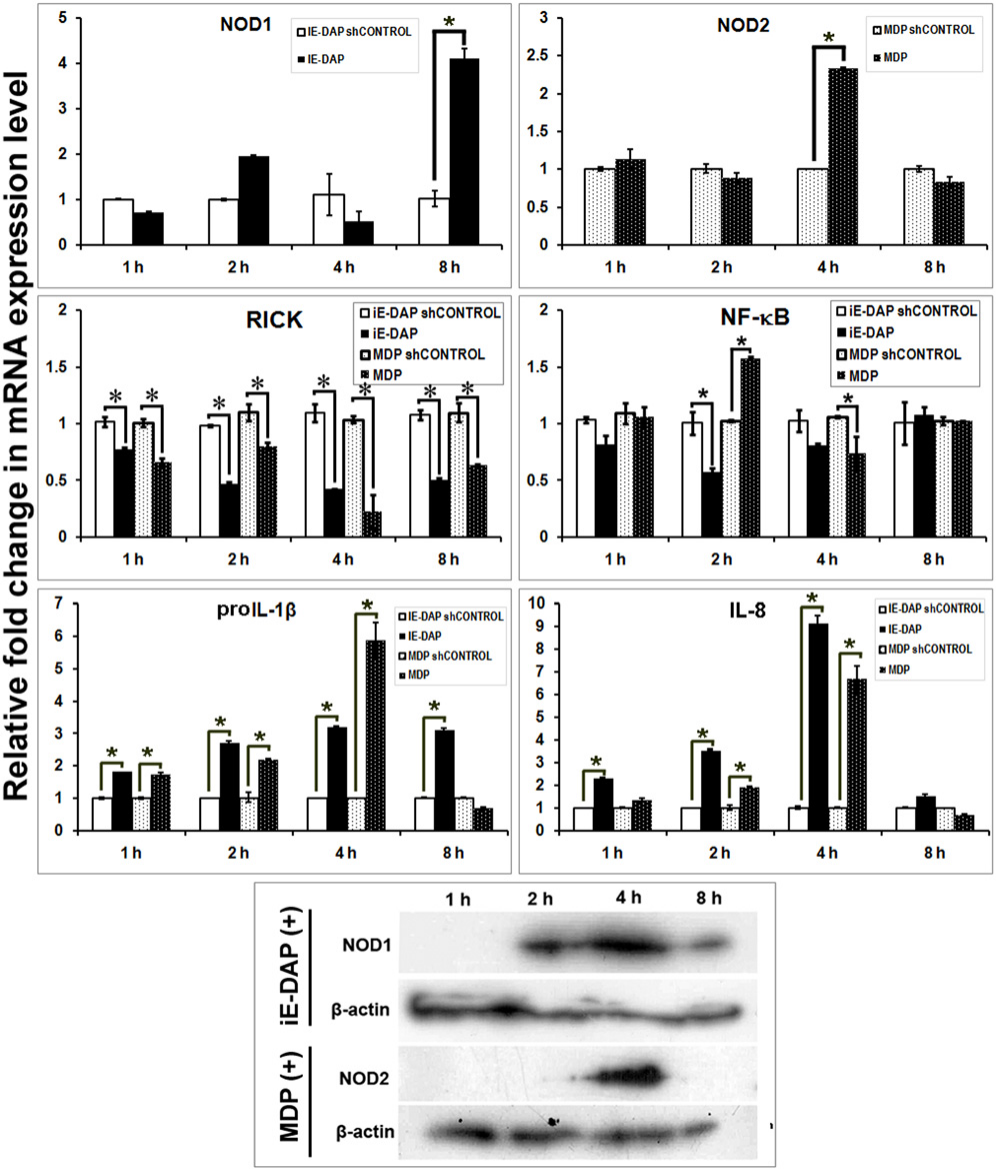
Inductive mRNA expression of NOD1, NOD2, interleukins and IFN-γ in iE-DAP and MDP treated alveolar macrophages of buffalo. X-axis represents the time intervals following agonists addition, Y axis shows relative fold change in mRNA expression of genes over respective controls (sh-controls). Columns indicated with asterisks (*) differ significantly (p<0.05) from their respective controls. Protein expression of NOD1, NOD2 and β-actin in treated cells over different time intervals has been shown also.

Alveolar macrophages showed increased NOD1 mRNA expression during 8 h post iE-DAP treatment (Fig. 5). Protein expression was evident during 2–8 h, reaching the peak during 4 h post treatment. Protein and mRNA expression of NOD2 were higher during 4 h post MDP treatment. The mRNA levels of RICK were consistently low throughout the experimental hours in both iE-DAP and MDP treated cells. NF-κB mRNA expression showed an increase during 2 h but later declined (4 h) in MDP treated macrophages. A pronounced pro-inflammatory cytokine response with elevated level of proIL-1β and IL-8 was observed in resident macrophages treated with NOD agonist. The levels of the cytokines increased as early as 1 h, attained maximal level during 4 h, and sustained up to 8 h in iE-DAP treated cells and up to 4 h in MDP treated cells. No significant expression of IFN-γ was observed in either treated or control cells. In brief, we found that both the peptidoglycans (iE-DAP and MDP) induced a pro-inflammatory cytokine response in alveolar macrophages.

### Agonist induced cellular response of buMECs

**Fig 6:**
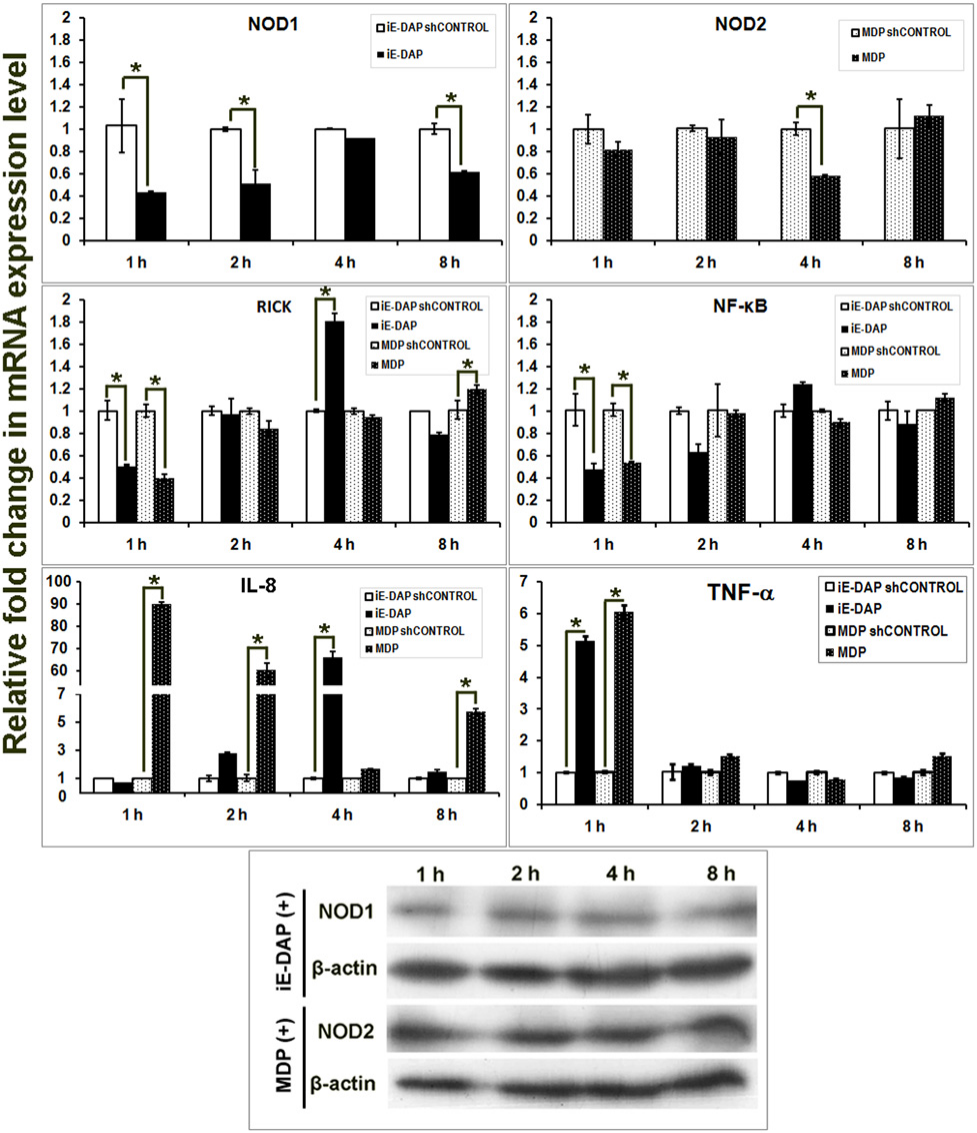
Inductive mRNA expression of NOD1, NOD2, RICK, NF-κB and IL-8 in iE-DAP and MDP treated mammary epithelial cell line of buffalo. X-axis represents the time intervals following agonist addition, Y axis shows relative fold change in mRNA expression of genes over respective controls (sh-controls). Columns indicated with asterisks (*) differ significantly (p<0.05) from their respective controls. Protein expression of NOD1, NOD2 and β-actin in treated cells over different time intervals has been shown also.

Mammary epithelial cells of buffalo, contrary to PBMC and macrophages, showed down-regulation of mRNA expression of NOD1 and NOD2 following agonist treatments (Fig. 6). However, notwithstanding with mRNA levels, coherent protein expression of NODs were observed during experimental hours. After initial down-regulation, RICK mRNA were significantly up-regulated following iE-DAP (4 h) and MDP (8 h) treatments. NF-κB mRNA expression declined initially and showed no increase in the levels during subsequent hours of experiment. However, immunocytochemical detection of NF-κB protein indicated nuclear translocation during 2–4 h in both iE-DAP and MDP treated cells (Table 1; S5 Fig.). Among pro-inflammatory cytokines, expression of IL-8 was higher in mammary epithelial cells compared to other cell types, but no appreciable level of proIL-1β was detected either in control or treated cells. Duration of elevated IL-8 expression was longer in MDP treated cells and was evident only during 4 h in iE-DAP treated cells. No detectable IFN-γ mRNA expression was observed in either control or treated mammary epithelial cells.

**Table 1:** Extent of NF-kB translocation to nucleus in different cell types following MDP and iE-DAP treatments.

Cell type	Agonist	Extent of translocation			
		1 h	2 h	4 h	8 h
Mammary epithelial cells	MDP	++	++++	+++	-
	IE-DAP	-	+++	++	-
Fibroblasts	MDP	+++	++++	++	-
	IE-DAP	+++	++++	-	-

### Agonist induced cellular response of buffalo foetal fibroblast cells

**Fig 7:**
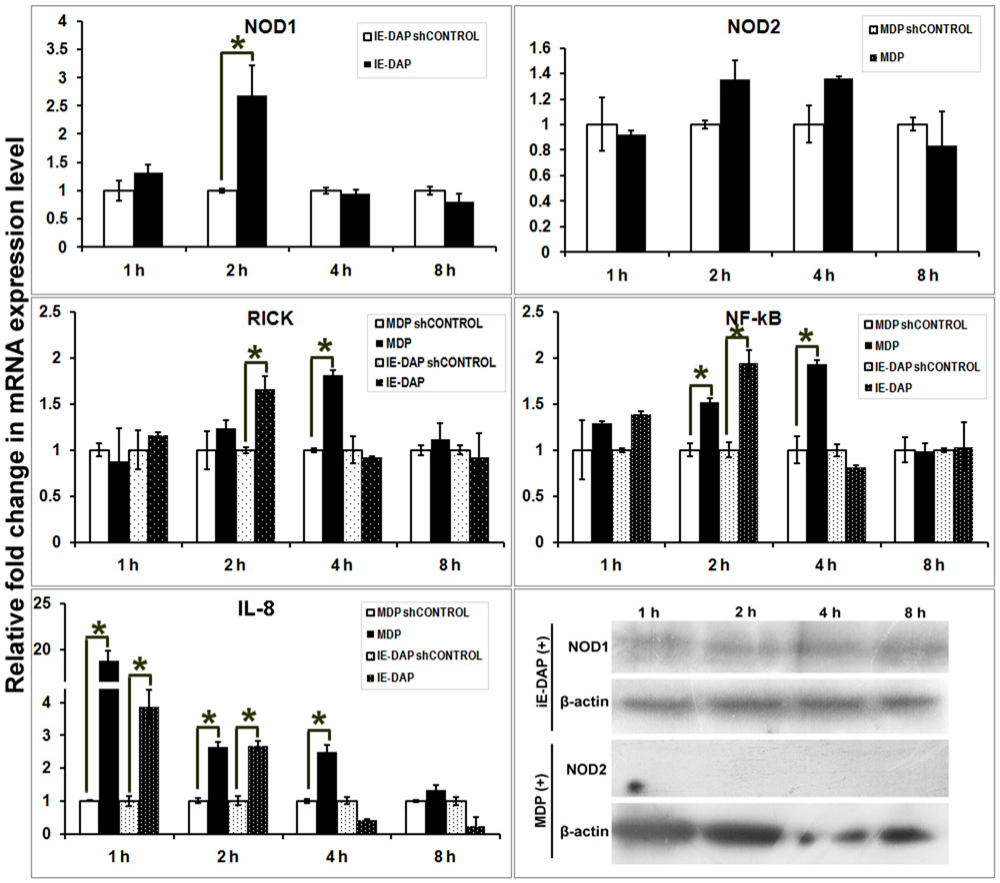
Inductive mRNA expression ofNOD1, NOD2, RICK, NF-κB and IL-8 in iE-DAP and MDP treated foetal fibroblast cells of buffalo. X-axis represents the time intervals following agonist addition, Y axis shows relative fold change in mRNA expression of genes over respective controls (sh-controls). Columns indicated with asterisks (*) differ significantly (p<0.05) from their respective controls. Protein expression of NOD1, NOD2 and β-actin in treated cells over different time intervals has been shown also.

In fibroblasts, expression of NOD mRNA or proteins were less apparent compared to other cell types. NOD1 mRNA expression increased during 2 h post iE-DAP treatment, but no significant increase of NOD2 mRNA was observed in MDP treated cells (Fig. 7). At protein level, lower NOD1 but no detectable NOD2 expressions were observed in fibroblast cells following agonist treatments. It was intriguing that despite little change in NOD2 mRNA or protein, expression of RICK mRNA was significantly higher during 4 h in MDP treated cells. Also, NF-κB mRNA expression showed a significant increase during 2–4 h post MDP treatment and it coincided with the period of NF-κB translocation to nucleus (Table 1; S6 Fig.). Concomitant expression of pro-inflammatory cytokine IL-8 was initialized early (1 h) and sustained up to 4 h in MDP treated fibroblasts. The mRNA expression of RICK and NF-κB were higher in iE-DAP treated cells during 2 h and accompanied increased IL-8 expression in fibroblast cells. Alike mammary epithelial cells, expressions of proIL-1β or IFN-γ were not found in peptidoglycan treated fibroblast cells.

## Discussion

The function and intracellular signaling mechanisms of action of NOD1 and NOD2 have been extensively studied in both immune and non-immune cells. Nevertheless, little is known about the genetic composition of these receptors and their role in innate immune response of buffalo. This study provides a systematic analysis of buNOD1 and buNOD2 genes and elucidates their role in immune response of cells following exposure to bacterial peptidoglycans.

Similarities in domain organization and physiological function of NOD1 and NOD2 are tempting to speculate that the genes had a common ancestor and one of them had evolved from the other by a gene duplication event. A high level of inter-species sequence conservation of NOD orthologs but consistently low homology between NOD1 and NOD2 among different species are notwithstanding with this hypothesis. Comparative analysis of the respective domains is also suggestive of independent evolutionary pattern of the two receptors. Buffalo shares a similar type of domain organization of NOD1 and NOD2 as found in other mammals. However, despite homology with mammalian orthologs, buNOD1 and buNOD2 have important genetic differences at sites implicated in binding of critical residues or signaling. For example, residues identified in human as ubiquitin binding sites during CARD-CARD interaction of NOD1/NOD2 and RICK, were different in buffalo. It could be possible that ubiquitinylation pattern is different in this species as homology modeling showed some of these designated ubiquitin binding sites were not accessible to solvent on the surface. Although the respective domains of buNOD1 and buNOD2 shared a little sequence identity, they shared analogy in the tertiary structures. Thus, despite difference in the primary sequences of the receptors, the helixes, turns, folds, and overall tertiary conformation of CARDs, NACHT, and LRR domains were of similar type. In addition, the pattern of residue conservation for ligand recognition at the concave face of LRRs showed remarkable resemblance. Together, this indicates a unified and evolutionary conserved mechanism(s) for ligand recognition and downstream signaling for the receptors. However, such interpretation warrants validation by crystallographic studies on ligand-receptor and receptor-adapter interactions of NOD1 and NOD2.

The constitutive expression pattern of NODs in different tissues suggested their potential function is not confined to immune organs. This suggests that both immune and non-immune cells have the potential for NLR mediated innate immune response to MAMP and DAMP signals. This assumption is supported by the fact that fibroblasts and mammary epithelial cells, in addition to PBMCs and macrophages, were able to initiate an immune response *in-vitro* following iE-DAP or MDP addition. However, it was evident that amplitude and duration of NOD mediated immune response varied in different cell types. This is reasonable, since mechanisms of antigen uptake, signaling and regulation of NOD pathways vary in different cell types. Information on uptake of NOD ligands by cells is sparse, but at least the process of MDP uptake is different for monocytes and epithelial cells. In monocytes, MDP internalization involves clathrin and dynamin-dependent endocytic pathway [66], which is slightly different in epithelial cells in that the apical peptide transporter PepT1 seems to have a role in delivery of MDP [67]. The distinctive IFN-γ response of buPBMCs following NOD stimulation indicates an alternative or additional signaling pathway with possible role of γδ T cells, as the response was specific to young calves with higher proportion of peripheral γδ T cells. With growing evidences, it appears that γδ T cells, besides harboring vast diversity of T cell receptors (TCRs) for recognition of peptide, phosphor and as non-peptide antigens [68–69], are also equipped with TLRs [70] and NODs [61] for sensing bacterial products or cell wall constituents. The cells constitutively express NOD2 mRNA and protein and up on MDP stimulation produce IFN-γ, a response more apparent in freshly isolated cells [61] but could be missing in *in vitro* expanded cells [62]. Similar observations have been reported with co-stimulation of TCR/TLR3 ligand [71] and the discrepancy could be attributed to different activation status of freshly isolated γδ T cells [61, 70]. γδ T cell activation and IFN- γ secretion are also influenced by other cytokines, including IL-12, IL-15, IL-18 and TNF-α [72–74]. Along with IFN-γ production, the cells also trigger a brief inflammatory response following MDP stimulation compared to typical prolonged responses of monocyte and macrophages [9, 62]. This was evident in this study as well, where pro-inflammatory cytokine response ceased to exist after 1 h of stimulation. This suggests different antigen delivery [62] and regulatory mechanisms contributes to MDP “tolerance” in γδ T cells. Recent studies have shown that cells induce tolerance to subsequent exposures of bacterial peptidoglycans to protect against detrimental consequences of excessive pro-inflammatory cytokine production [75–76]. MDP tolerance in macrophages could be initiated as early as 4 h and is mediated by dissociation of chaperon protein Hsp90 from NOD2 and subsequent ubiquitination and rapid degradation of NOD2 [76]. However, development of such tolerance necessitates pre-exposure to MDP and is regulated at the protein level rather than at transcript level [76]. Regulation of protein expression independent of transcript level also could account for unsynchronized expression pattern of NODs mRNA and proteins, observed in this study and earlier [24, 76]. While absence of a MDP pre-exposure justifies a prolonged inflammatory response of alveolar macrophages found in this study, the decline of both NOD2 and RICK mRNA levels in buPBMCs during 4 h of experiment suggest the prospect of regulation of an overt inflammatory response at transcript level. Alternatively, this decline could be associated with a high IFN-γ level, as priming of macrophages with IFN- γ has been shown to reduce NOD2 transcripts [76].

The *in-vivo* environment of mammary epithelial cells is not strictly sterile, and therefore it is not surprising that cells are capable of TLR and NOD mediated immune response as found in this study and in earlier reports [77–78]. However, contrary to the earlier report [56], a pro-inflammatory cytokine response was shown by buMECs. The response of fibroblast during initial hours of MDP treatment was interesting as increased RICK, NF- κB translocation, and IL-8 production were observed without traceable NOD2 protein expression. Possible involvement of TLR2, TLR2/1 or TLR2/6 associations can be excluded here for two reasons. First, it is now established that MDP specifically activates NOD2 and is not recognized by TLRs [13, 79]. Second, only NOD1/NOD2 can induce RICK/RICK mediated NF-κB translocation but this is not feasible with TLRs [16]. A probable explanation could be that MDP uptake and subsequent signaling are NOD2-independent in apparently NOD2 deficient fibroblast cells. MDP internalization could be mediated through pannexin-1, followed by recognition and binding with NLRP3 that leads to activation of caspase-1 [80]. MDP stimulation through NLRP3 pathway however, requires NOD2 for pro-IL-1β production as the response was abolished in NOD2-deficient macrophages [80]. This explains lack of pro-IL-1β response with fibroblasts in this study with no detectable expression of NOD2. An alternative viewpoint is that sensitization of fibroblast for RICK/RICK mediated NF-κB signaling occurs at very low NOD2 concentration and overproduction is prevented by a stringent regulation. Together, the study demonstrated that many cell types have the potential for NOD mediated immune response though the responsiveness of NODs to peptidoglycan and the mode of immune response could vary in different cell types.

## Conclusion

Comparative genomic analysis of NOD1 and NOD2 suggested an inter-species conservation of the orthologous sequences and analogy in the tertiary structures of respective domains of the receptors. The functional roles of the receptors are not confined to immune organs as non-immune cells, such as fibroblasts and epithelial cells, also showed *in-vitro* NOD-mediated immune response. However, the mode of immune response is not uniform in different cell types.

## Supporting Information

S1 Fig Specificity of NOD1 and NOD2 antibodies as determined by western blot. Tissue lysate of spleen was probed with the antibodies at 1:200 dilution.
(TIF)

S2 Fig Repertoire and evolutionary perspective of NLRs. Three building blocks of NLRs, an N-terminal DEATH folds, the central nucleotide binding domain (NB-ARC/NACHT domain), and a C-terminal LRR (leucine-rich repeats) domain have been identified in prokaryotes and lower eukaryotes. Fusion of domains (NACHT—LRR; NB-ARC—LRRs) had occurred independently in the early history of metazoans probably coinciding with the appearance of multicellularity. NLRs with tripartite domain organization (DED/CARD—NACHT—LRRs) have been identified in primitive animals like *Strongylocentrotus purpuratus* and cnidarians *Acropora digitifera*, although *Hydra magnipapillata* appears to lack the *bona fide* NLRs and instead has abundance of DEATH folds—NACHT domains containing genes. Teleost fish like *Danio rerio* has vast repertoire of NLRs with more than 70 human NLRC3 orthologous, true orthologs of NOD1 and NOD2, but no orthologs of human IPAF and NAIP. NLRs appear to be missing in few invertebrates like *Drosophila melanogaster* and *Caenorhabditis elegans*, while the NOD2 has been selectively lost in amphibian, birds and lizards. So far we have identified eight NLR genes with orthologs of NLRB, NLRCs and NLRPs in buffalo, but total number of NLRs in buffalo is likely to be higher.
(TIF)

S3 Fig Multiple sequence alignment of NACHT domains of NOD1 and NOD2. Residues and putative conserved motifs important for ATP binding have been shown. Secondary structure predicted by PSIPRED showed that NACHT domains of NOD1 and NOD2 were of similar kind but not identical.
(TIF)

S4 Fig mRNA expression levels of ATG-16L1 and other autophagy related genes in MDP treated PBMCs of buffalo. X-axis represents the time intervals following MDP addition, Y axis shows relative fold change in mRNA expression of genes over respective controls (sh-controls). No significant difference was observed in expression level of any of the gene following treatment.
(TIF)

S5 Fig Representative fluorescent micrographs of cells showing translocation of NF-κB to nucleus in iE-DAP and MDP treated mammary epithelial cells. Cells treated with sh-controls showed a hollow zone around nucleus indicating no translocation. Immunostaining of cells with β-actin showed that there was no problem with the staining procedure.
(TIF)

S6 Fig Representative fluorescent micrographs of cells showing translocation of NF-κB to nucleus in iE-DAP and MDP treated foetal fibroblast cells. Cells treated with sh-controls showed very faint staining spanning all over the cells. Immunostaining of cells with β-actin has also been shown.
(TIF)

S1 Table Primers used in this study for amplification of NOD1 and NOD2 genes. (DOCX)

S2 Table Primers used in this study for relative quantitation of mRNA by real time PCR. (DOCX)

S3 Table PCR cycling parameters used for amplification of NOD1 and NOD2 genes. (DOCX)

S4 Table Sequences used for comparative evolutionary analysis. (DOCX)

S5 Table Model validation scores depicting accuracy of stereochemical and overall quality parameters for different domains of NOD1 and NOD2. (DOCX)
