# Supplementary figures and images for: Comparative Genomic Analysis of Buffalo (Bubalus bubalis) NOD1 and NOD2 Receptors and Their Functional Role in In-Vitro Cellular Immune Response
Source: PLoS One. 2015 Mar 18;10(3):e0119178. doi: 10.1371/journal.pone.0119178 (PMC4365024; doi:10.1371/journal.pone.0119178)

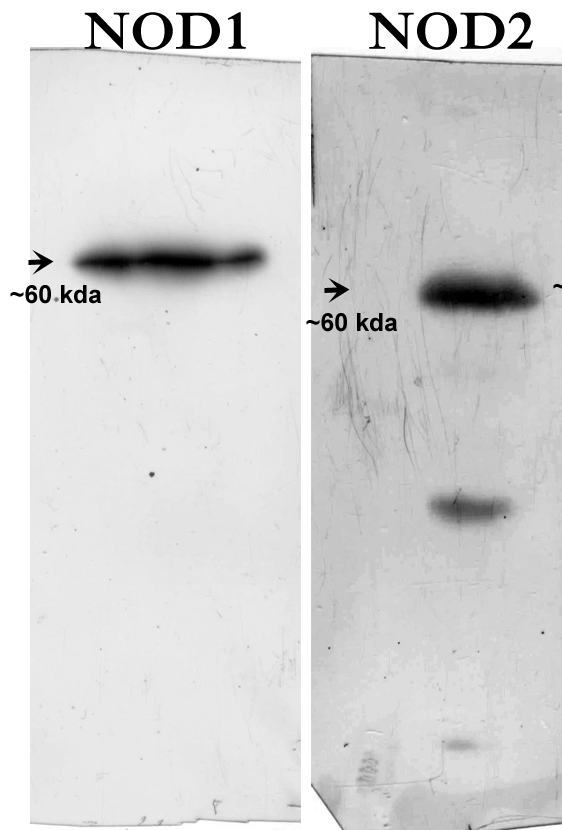

Supplement: S1 Fig — Tissue lysate of spleen was probed with the antibodies at 1:200 dilution. (TIF) [file pone.0119178.s001.tif]

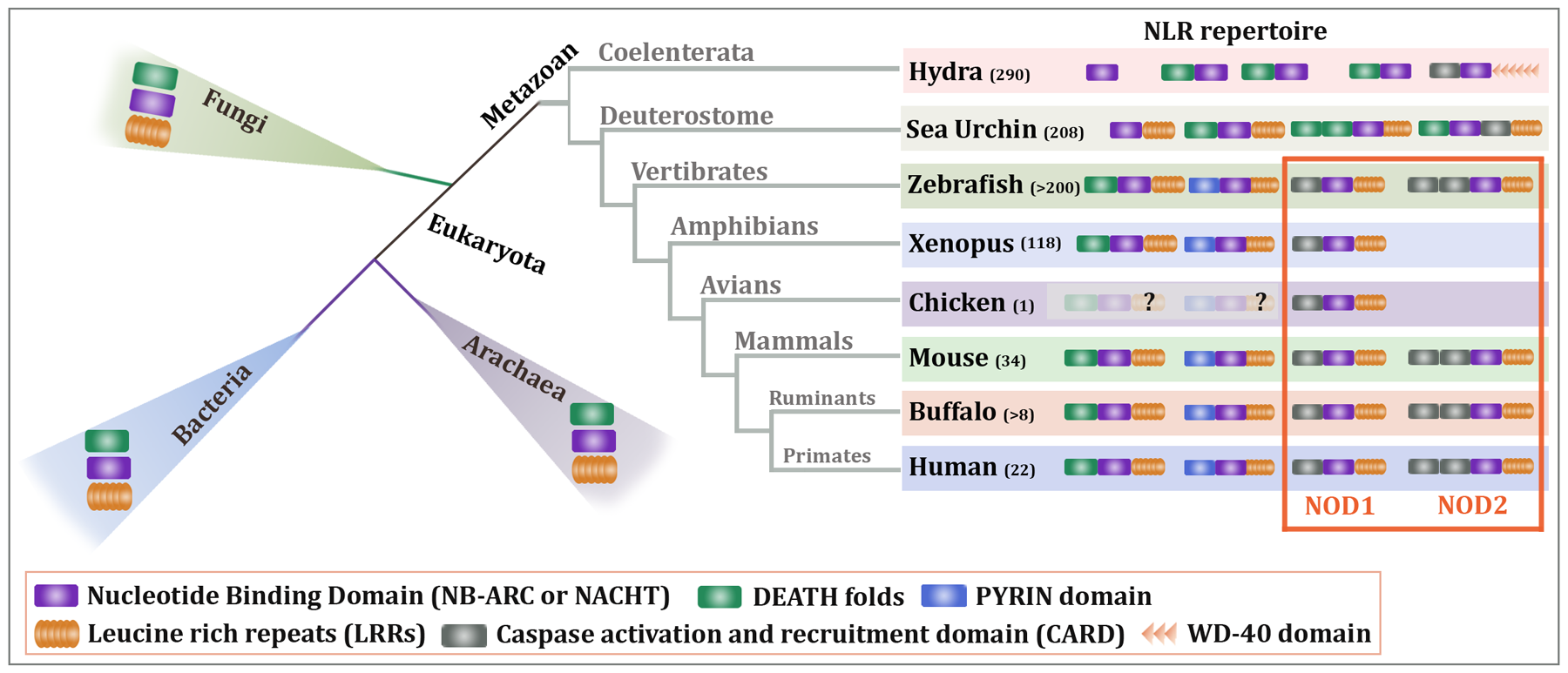

Supplement: S2 Fig — Three building blocks of NLRs, an N-terminal DEATH folds, the central nucleotide binding domain (NB-ARC/NACHT domain), and a C-terminal LRR (leucine-rich repeats) domain have been identified in prokaryotes and lower eukaryotes. Fusion of domains (NACHT—LRR; NB-ARC—LRRs) had occurred independently in the early history of metazoans probably coinciding with the appearance of multicellularity. NLRs with tripartite domain organization (DED/CARD—NACHT—LRRs) have been identified in primitive animals like Strongylocentrotus purpuratus and cnidarians Acropora digitifera, although Hydra magnipapillata appears to lack the bona fide NLRs and instead has abundance of DEATH folds—NACHT domains containing genes. Teleost fish like Danio rerio has vast repertoire of NLRs with more than 70 human NLRC3 orthologous, true orthologs of NOD1 and NOD2, but no orthologs of human IPAF and NAIP. NLRs appear to be missing in few invertebrates like Drosophila melanogaster and Caenorhabditis elegans, while the NOD2 has been selectively lost in amphibian, birds and lizards. So far we have identified eight NLR genes with orthologs of NLRB, NLRCs and NLRPs in buffalo, but total number of NLRs in buffalo is likely to be higher. (TIF) [file pone.0119178.s002.tif]

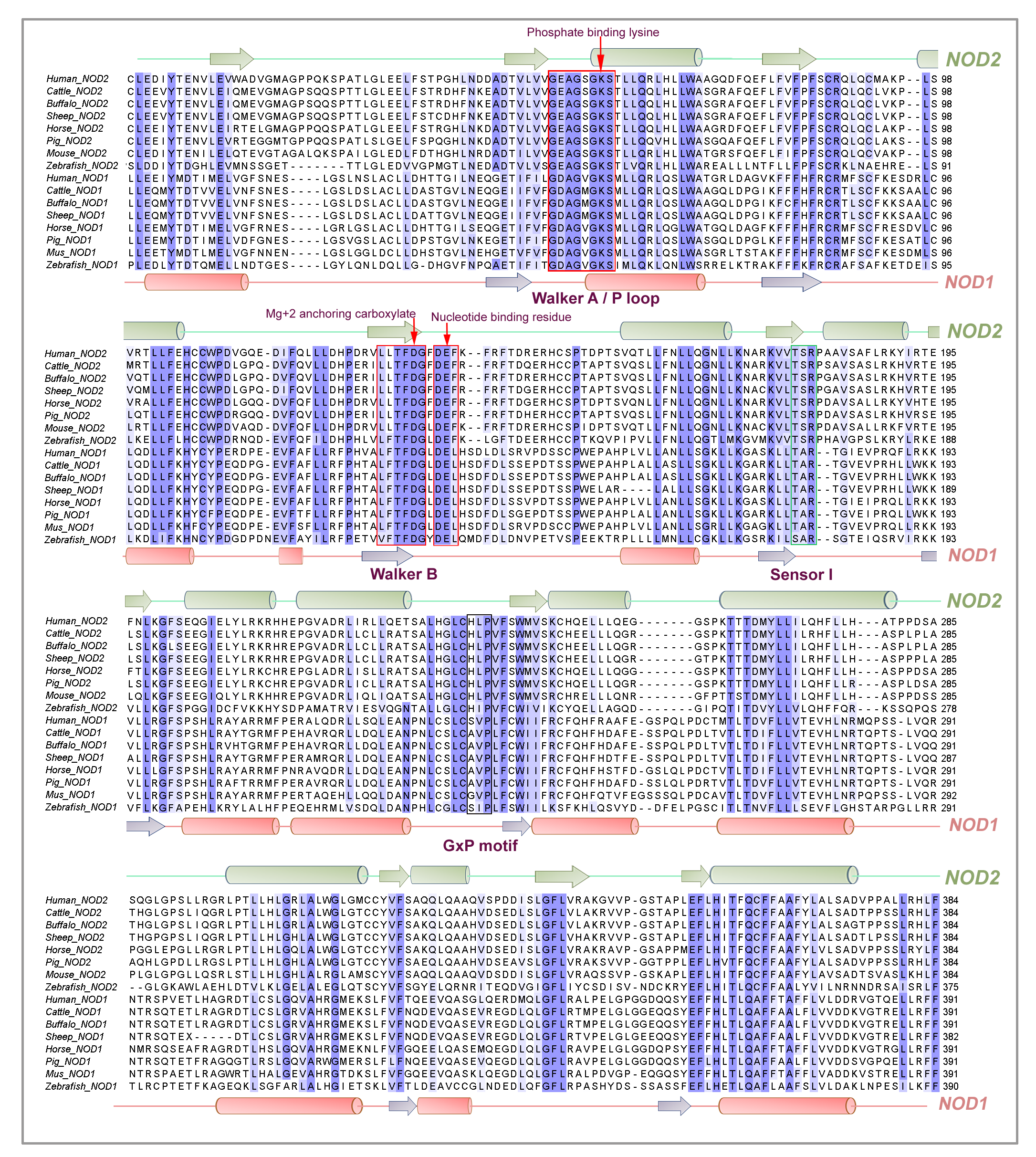

Supplement: S3 Fig — Residues and putative conserved motifs important for ATP binding have been shown. Secondary structure predicted by PSIPRED showed that NACHT domains of NOD1 and NOD2 were of similar kind but not identical. (TIF) [file pone.0119178.s003.tif]

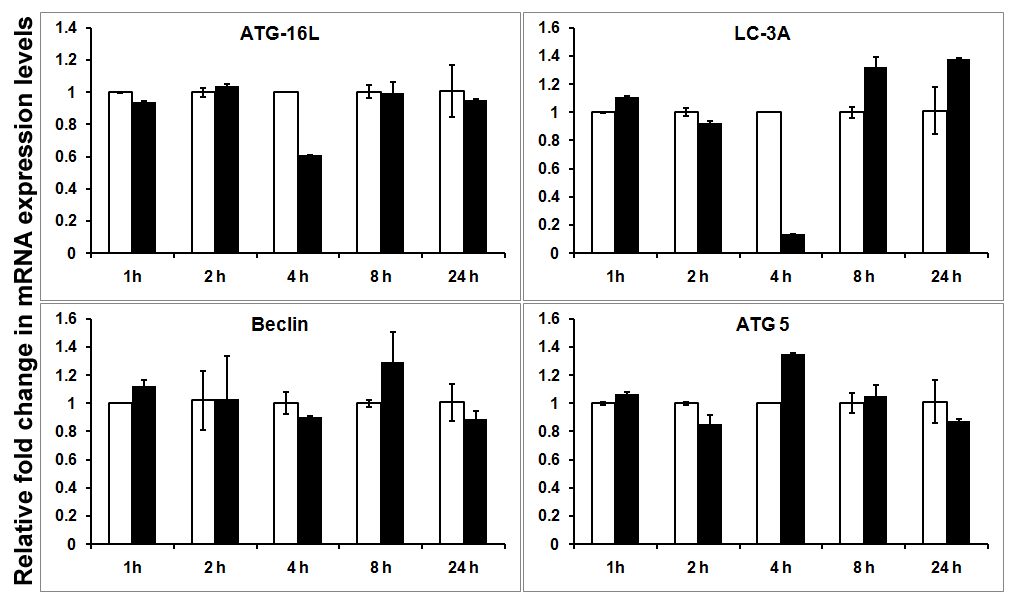

Supplement: S4 Fig — X-axis represents the time intervals following MDP addition, Y axis shows relative fold change in mRNA expression of genes over respective controls (sh-controls). No significant difference was observed in expression level of any of the gene following treatment. (TIF) [file pone.0119178.s004.tif]

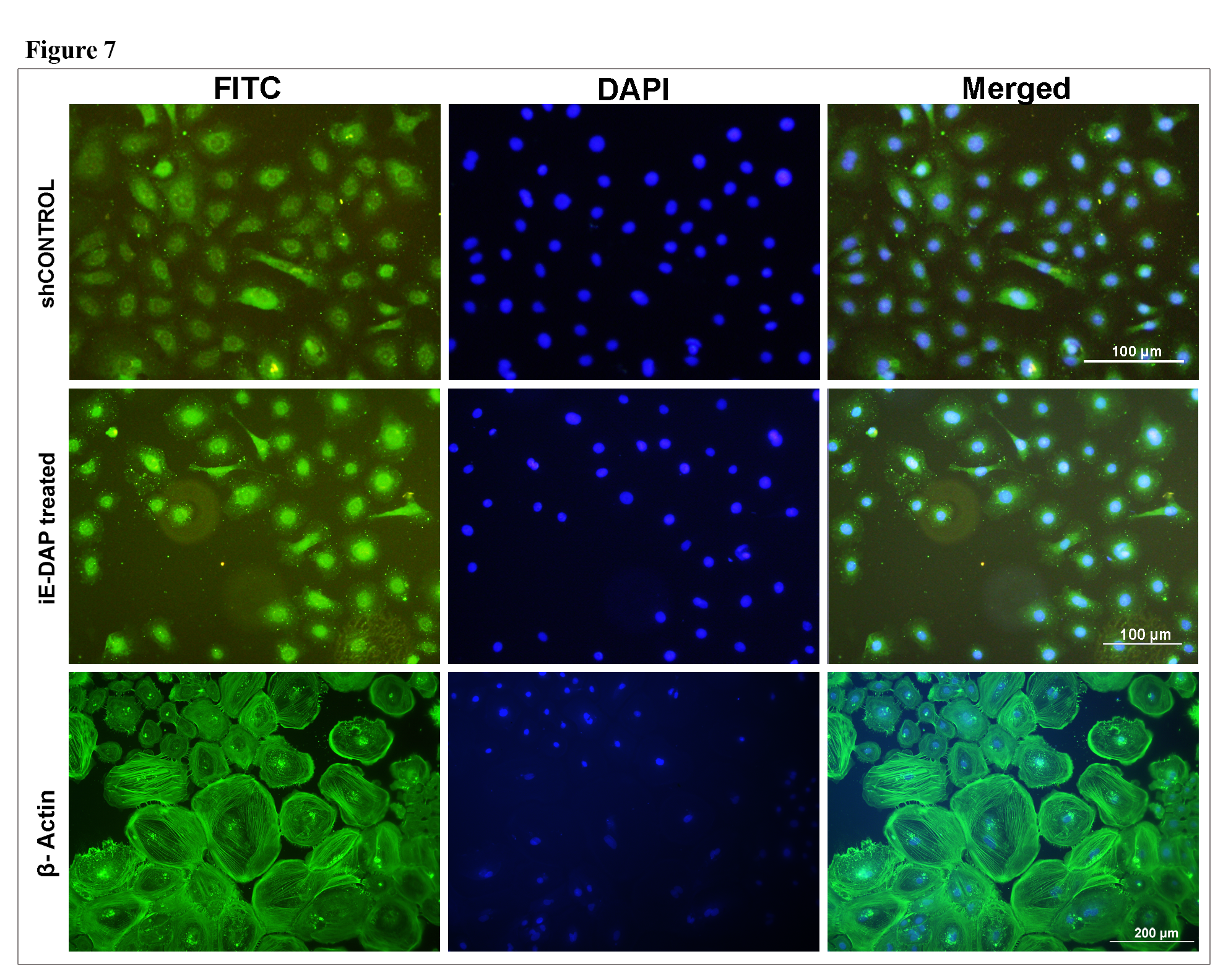

Supplement: S5 Fig — Cells treated with sh-controls showed a hollow zone around nucleus indicating no translocation. Immunostaining of cells with β-actin showed that there was no problem with the staining procedure. (TIF) [file pone.0119178.s005.tif]

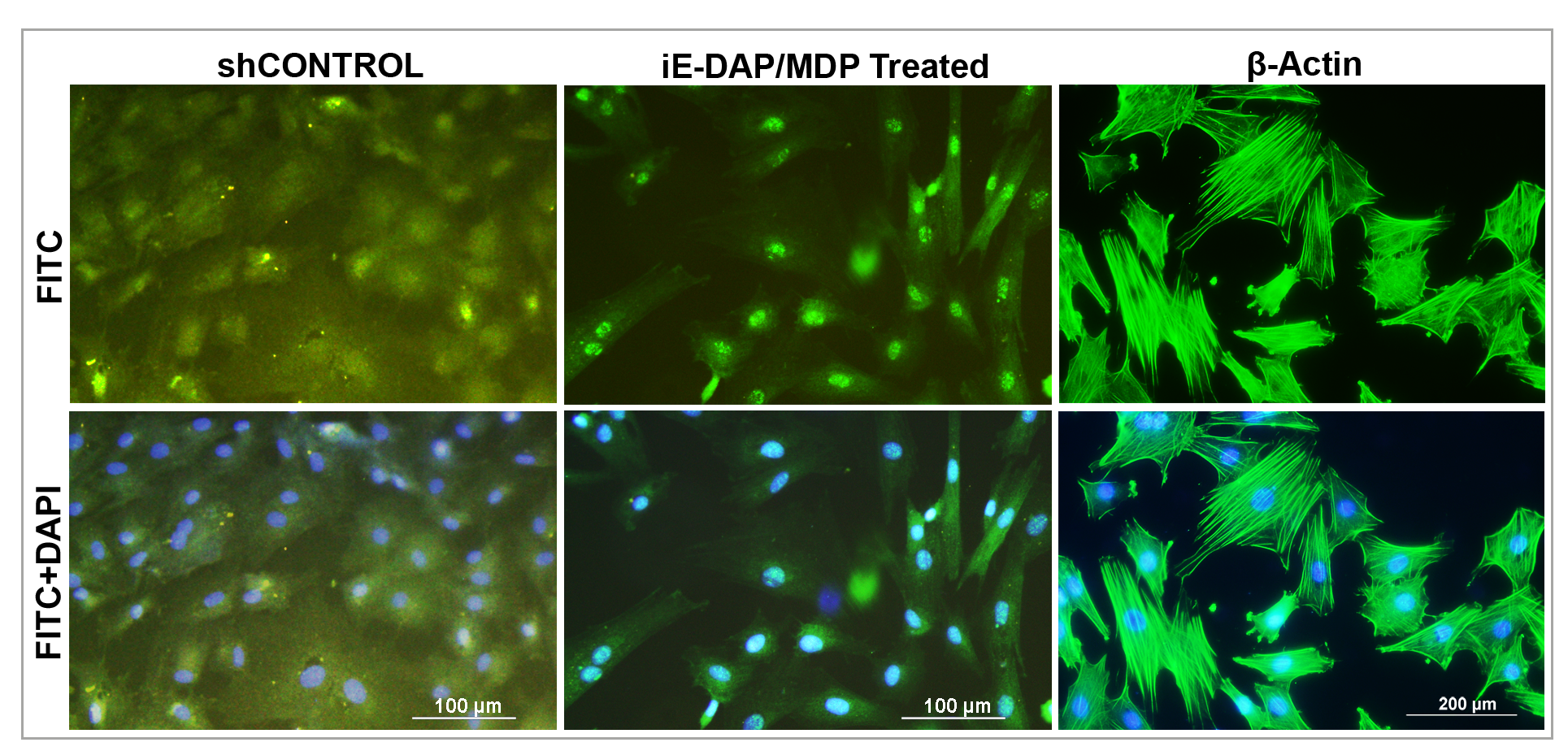

Supplement: S6 Fig — Cells treated with sh-controls showed very faint staining spanning all over the cells. Immunostaining of cells with β-actin has also been shown. (TIF) [file pone.0119178.s006.tif]
