# Supplementary material for: Comparative Genomic Analysis of Buffalo (Bubalus bubalis) NOD1 and NOD2 Receptors and Their Functional Role in In-Vitro Cellular Immune Response
Source: PLoS One. 2015 Mar 18;10(3):e0119178. doi: 10.1371/journal.pone.0119178 (PMC4365024; doi:10.1371/journal.pone.0119178)
Supplement: S1 Table — (DOCX) [file pone.0119178.s007.docx]

| **Gene** | **Frag.** | **Primer name** | **Sequence (5′ – 3′)** | **T_m_ (^0^C)** | **Length**  **(bp)** |
| --- | --- | --- | --- | --- | --- |
| *NOD1* | I | NOD1FR1FW | ATGGAAAAGCACGGCTGCAGTAAGATGG | 28 | 61 |
|  |  | NOD1FR1RV | CACCGGAAGATGATCCAGCAGAAGAG | 26 | 61 |
|  | II | NOD1FR2FW | AACCCCAACCTCTGCAGCCTGTGC | 24 | 63 |
|  |  | NOD1FR2RV | GTCTCGTAGATGCAGCGCAACATCCA | 26 | 61 |
|  | III | NOD1FR3FW | TTCACCAACCTCTTCCTGTGCGGGCT | 26 | 63 |
|  |  | NOD1FR3RV | CTTCTCATCTTCAAAGACCTTGGCCTCCTC | 30 | 63 |
| *NOD2* | I | NOD2FR1FW | ATTGTGAAATGTGCGCACAAGATGCTTTTCAG | 32 | 61 |
|  |  | NOD2FR1RV | CGGCAGCTAAATGGGAAGACGAAGAG | 26 | 61 |
|  | II | NOD2FR2FW | GCAGACACTGTGCTGGTGGTGGG | 23 | 62 |
|  |  | NOD2FR2RV | CTGTGATCTGGAGGTTGTGCGGCTC | 25 | 63 |
|  | III | NOD2FR3FW | CTGCATTCTACCTCGCCCTCAGTGC | 25 | 63 |
|  |  | NOD2FR3RV | GGAAACATCAGAGCAAGAGTCTGGTATCC | 29 | 62 |

**Table S1: Primers used in this study for amplification of NOD1 and NOD2 genes**
