# Supplementary material for: Comparative Genomic Analysis of Buffalo (Bubalus bubalis) NOD1 and NOD2 Receptors and Their Functional Role in In-Vitro Cellular Immune Response
Source: PLoS One. 2015 Mar 18;10(3):e0119178. doi: 10.1371/journal.pone.0119178 (PMC4365024; doi:10.1371/journal.pone.0119178)
Supplement: S2 Table — (DOCX) [file pone.0119178.s008.docx]

**Table S2: Primers used in this study for relative quantitation of mRNA by real time PCR**

| **Gene** | **Primer name** | **Sequence (5′ – 3′)** | **T_m_ (^0^C)** | **Length**  **(bp)** |
| --- | --- | --- | --- | --- |
| *NOD1* | NOD1RTFW | CGTCAGACTCAGTGTGAACCAGATCAC | 61 | 27 |
|  | NOD1RTRV | GGCTCCGACATCGGTGATTTGGTTGTTG | 63 | 28 |
| *NOD2* | NOD2RTFW | GTGACCTGCAGAGTCACCGACCAGC | 64 | 25 |
|  | NOD2RTRV | AAAGGCAGCCAACCCATTCGCCTTCAC | 63 | 27 |
| *NFKB* | NFKBRTFW | ATCCACCTGCATGCACACAGCCTGGTG | 64 | 27 |
|  | NFKBRTRV | CAGGCATCTGTCATTCGTGCTTCCAGTGTTT | 63 | 31 |
| *IL10* | IL10RTFW | TTCCTCTTTGGCCTTATAAACCAGGAGAG | 63 | 26 |
|  | IL10RTRV | AGGGCAGAAAGCGATGACAGCGCCG | 64 | 25 |
| *IL-8* | IL08RTFW | TTGGCAGCTTTCCTGCTCTCTGCAGCTCT | 64 | 29 |
|  | IL08RTRV | CTGAATTTTCACAGTGTGGCCCACTCTC | 61 | 28 |
| *IL1B* | IL1BRTFW | GGTGTTCTGCATGAGCTTTGTGCAAGGAGA | 63 | 30 |
|  | IL1BRTRV | CCTCTTGGGGTAGACTTTGGGGTCTA | 61 | 26 |
| *GMCSF* | GMCSFRTF | CCCTGGCAGCATGTGGATGCCATCAA | 63 | 26 |
|  | GMCSFRTR | TTGTACAGCTTCAGGCGAGTCTGCAGG | 63 | 27 |
| *RICK* | RICKRTF | GCCGTGAAGCACCTGCACATCCACA | 63 | 25 |
|  | RICKRTR | CAGGCTCATTGCAAATTCCCAAAATTGGAAGA | 61 | 32 |
| *IFNG* | IFNGRTF | CAAAGGAGCATGGATATCATCAAGCAAGACATG | 62 | 33 |
|  | IFNGRTR | CTTTGATGAGTTCATTTATGGCTTTGCGCTGG | 62 | 32 |
| *RPS18R* | RPS18RTFW | TGCGAGTACTCAACACCAACATCGATGG | 61 | 28 |
|  | RPS18RTRV | GGATTCTGCATAATGGTGATCACACGTTCC | 62 | 30 |
| *BECN* | BECN1RTFW | AGCACCATGCAGGTGAGCTTCGTGTG | 63 | 26 |
|  | BECN1RTRV | ATGAATCTGCGAGAGACACCATCCTGGC | 63 | 28 |
| *ATG5* | ATG5RTFW | AGATGTGCTTCGAGATGTGTGGTTTGGACG | 63 | 30 |
|  | ATG5RTRV | GCCATTTCAGTGGTGTGCCTTCATATTCAAACC | 63 | 33 |
| *LC3A* | LC3ARTFW | CTGCCGGTCCTGGACAAGACCAAGTT | 63 | 26 |
|  | LC3ARTRV | GAAGGTTTCCTGGGAGGCGTAGACCAT | 63 | 27 |
| *ATG16L1* | ATG16RTFW | CAAGTTGCTGGAAAAGTCAGATCTTCATTCAGT | 61 | 33 |
|  | ATG16RTRV | CTGCTGCATTTGGTTATTCAGGTCAATCACCA | 63 | 32 |
| *TNFA* | TNFARTFW | ACCACGCTCTTCTGCCTGCTGCACT | 63 | 25 |
|  | TNFARTRV | TCAGCAGGCACCACCAGCTGGTTGTCT | 63 | 27 |
