# Supplementary material for: Comparative Genomic Analysis of Buffalo (Bubalus bubalis) NOD1 and NOD2 Receptors and Their Functional Role in In-Vitro Cellular Immune Response
Source: PLoS One. 2015 Mar 18;10(3):e0119178. doi: 10.1371/journal.pone.0119178 (PMC4365024; doi:10.1371/journal.pone.0119178)
Supplement: S3 Table — (DOCX) [file pone.0119178.s009.docx]

**Table S3: PCR cycling parameters used for amplification of NOD1 and NOD2 genes**

| **Step** | **Process** | **Temperature (^0^C)** | **Time** |
| --- | --- | --- | --- |
|  | Initial denaturation | 94 | 5 min |
|  | Denaturation | 94 | 30s |
|  | Annealing | 64; Δt = 1^0^C/cycle | 30s |
|  | Extension | 68 | 1 min |
|  | Repeat steps 2-4 (5 cycles) | | |
|  | Denaturation | 94 | 30s |
|  | Annealing | 58 | 30s |
|  | Extension | 68 | 1 min |
|  | Repeat steps 6-8 (30 cycles) | | |
|  | Final Extension | 68 | 5 min |
|  | Holding | 4 | ∞ |
