# Supplementary material for: Comparative Genomic Analysis of Buffalo (Bubalus bubalis) NOD1 and NOD2 Receptors and Their Functional Role in In-Vitro Cellular Immune Response
Source: PLoS One. 2015 Mar 18;10(3):e0119178. doi: 10.1371/journal.pone.0119178 (PMC4365024; doi:10.1371/journal.pone.0119178)
Supplement: S4 Table — (DOCX) [file pone.0119178.s010.docx]

**Table S4: Sequences used for comparative evolutionary analysis**

| Species | Protein Accession ID | |
| --- | --- | --- |
|  | NOD1 | NOD2 |
| *Homo sapiens* | NP_006083.1 | NP_071445.1 |
| *Bos taurus* | NP_001243492.1 | NP_001002889.1 |
| *Capra hircus* | XP_005679309.1 | XP_005692135.1 |
| *Ovis aries* | XP_004007979.1 | XP_004015029.1 |
| *Equus caballus* | XP_001499616.1 | XP_001915323.2 |
| *Sus scrofa* | NP_001107749.1 | NP_001098765.1 |
| *Mus musculus* | NP_766317.1 | NP_665856.2 |
| *Danio rerio* | XP_002665106.3 | XP_697924.3 |
