# Supplementary material for: Comparative Genomic Analysis of Buffalo (Bubalus bubalis) NOD1 and NOD2 Receptors and Their Functional Role in In-Vitro Cellular Immune Response
Source: PLoS One. 2015 Mar 18;10(3):e0119178. doi: 10.1371/journal.pone.0119178 (PMC4365024; doi:10.1371/journal.pone.0119178)
Supplement: S5 Table — (DOCX) [file pone.0119178.s011.docx]

| **Domain** | **Ramachandran plot %** | **G-factor** | **Verify 3D %** | **Errat** | **ProSA** | **ProQ** |
| --- | --- | --- | --- | --- | --- | --- |
| buNOD2-CARDa | Most favoured : 92.3  Additional allowed : 7.7  Generously allowed : 0.0  Disallowed : 0.0 | -0.08 | 85.56 | 60.494 | -6.18 | LGscore : 5.895 MaxSub : 0.940 |
| buNOD2-CARDb | Most favored : 90.9  Additional allowed : 6.5  Generously allowed : 1.3  Disallowed : 1.3 | -0.18 | 85.56 | 61.728 | -5.02 | LGscore: 4.042  MaxSub: 0.892 |
| bu_NOD1-CARD | Most favored : 84.7  Additional allowed : 8.2  Generously allowed : 5.9  Disallowed : 1.2 | -0.14 | 85.22 | 79.518 | -3.15 | LGscore: 4.249  MaxSub: 0.819 |
| bu_NOD1_NATCH | Most favored : 88.9  Additional allowed : 8.9  Generously allowed : 1.3  Disallowed : 1.0 | -0.17 | 81.01 | 60.947 | -5.58 | LGscore: 5.390  MaxSub: 0.963 |
| bu_NOD2_NATCH | Most favored : 91.5  Additional allowed : 5.7  Generously allowed : 1.4  Disallowed : 1.4 | -0.06 | 85.82 | 63.503 | -4.23 | LGscore: 5.832  MaxSub: 0.918 |
| bu_NOD1_LRR | Most favored : 80.9  Additional allowed : 16.5  Generously allowed : 1.5  Disallowed : 1.1 | -0.2 | 87.68 | 69.303 | -3.99 | LGscore: 4.603  MaxSub: 0.759 |
| bu_NOD2_LRR | Most favored : 79.6  Additional allowed : 17.6  Generously allowed : 1.2  Disallowed : 1.5 | -0.2 | 87.31 | 70.1 | -4.18 | LGscore: 6.460  MaxSub: 0.696 |

**Table S5: Model validation scores depicting accuracy of stereochemical and overall quality parameters for different domains of NOD1 and NOD2**
